# Unconventional secretion of annexins and galectins

**DOI:** 10.1016/j.semcdb.2018.02.022

**Published:** 2018-11

**Authors:** Stephanie J. Popa, Sarah E. Stewart, Kevin Moreau

**Affiliations:** University of Cambridge, Metabolic Research Laboratories, Wellcome Trust-Medical Research Council Institute of Metabolic Science, Cambridge, CB2 0QQ, UK

**Keywords:** Unconventional secretion, Galectins, Annexins, Transport

## Abstract

Eukaryotic cells have a highly evolved system of protein secretion, and dysfunction in this pathway is associated with many diseases including cancer, infection, metabolic disease and neurological disorders. Most proteins are secreted using the conventional endoplasmic reticulum (ER)/Golgi network and as such, this pathway is well-characterised. However, several cytosolic proteins have now been documented as secreted by unconventional transport pathways. This review focuses on two of these proteins families: annexins and galectins. The extracellular functions of these proteins are well documented, as are associations of their perturbed secretion with several diseases. However, the mechanisms and regulation of their secretion remain poorly characterised, and are discussed in this review.

*This review is part of a Special Issues of SCDB on ‘unconventional protein secretion’ edited by Walter Nickel and Catherine Rabouille*.

## 1 Introduction

The conventional protein secretion pathway is now well-understood: proteins are directed into the endoplasmic reticulum (ER) lumen, or are anchored in its membrane, by an N-terminal signal sequence or hydrophobic segment. From here, proteins are exported out of the ER to the Golgi apparatus and are then trafficked to the cell surface via membrane-bound vesicles. Reviews of this pathway provide further details [[1], [2], [3], [4]]. However, not all secreted proteins use this pathway. A growing list of unconventionally secreted proteins have been described which do not have an N-terminal signal sequence yet are still functional outside of the cell [[5], [6], [7]]. Furthermore, their export is not blocked by inhibitors of the classical secretion pathway such as brefeldin A, showing that they do not use the classical ER-Golgi-plasma membrane pathway. The existence of this unconventional route for secretion may help in preventing inappropriate interactions. For example, glycoproteins and their ligands should be kept apart at some stages of trafficking to stop them interacting at the wrong time or place.

**Fig. 1:**
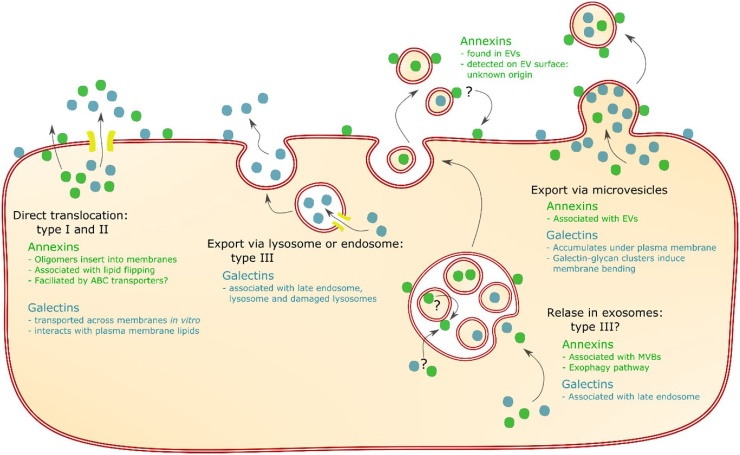
Pathways for unconventional secretion of galectins and annexins. Direct translocation, which may be facilitated or unfacilitated as part of type I secretion, or facilitated by ABC transporters in type II secretion. Export via lysosomes or endosomes, an example of type III secretion. Export in EVs, either via exosomes or via microvesicles; export via exosomes might be an example of type III secretion. It is unclear where annexin found on the surface of EVs originates from. It could come from crossing a membrane in MVBs, and might be transferred from EVs back to the plasma membrane, as indicated by arrows marked with question marks. Brief summaries of the evidence for each protein family using that pathways is shown for annexin in green and for galectin in blue.

Multiple pathways have been proposed to explain the mechanisms by which these unconventionally secreted proteins leave the cell, which fall into four categories. The three of these that will be discussed in this review are shown in Fig. 1. All four types involve crossing a membrane, with nomenclature as described by Rabouille et al. [8] For types I and II, proteins are directly translocated across the plasma membrane. Type I refers to translocation across the plasma membrane; either aided by other protein complexes, transported through pores, or unfacilitated. In type II secretion, translocation is via ATP-binding-cassette (ABC) transporter proteins. Type III describes the secretion of cytoplasmic proteins that first enter the lumen of an organelle, which then fuses with the plasma membrane. In type IV secretion, transmembrane proteins are inserted in the ER membrane, but reach the plasma membrane without passing through the Golgi.

In addition, cytoplasmic proteins can be released in extracellular vesicles (EVs), which are categorised as exosomes or microvesicles according to their origins [9]. Exosomes are formed from multivesicular bodies (MVBs) via invagination of the MVB membrane which creates internal vesicles – exosomes – that are loaded with cytoplasmic proteins. Upon fusion of MVBs with the plasma membrane, exosomes are released. This may be another example of type III secretion, although it is unclear if proteins reach the lumen of MVBs or remain inside exosomes. The second type of EVs are microvesicles, which derive from direct budding from the plasma membrane. Pathways involving EVs do not fully explain how an unconventional protein might be secreted; although proteins are outside the cell, they should remain inside vesicles, as shown in Fig. 1.

This review will focus on the unconventional secretion of two protein families: annexins and galectins. For both of these protein families, the presence of family members outside the cell has been documented. Annexin A1, A2, A4 and A5 are all found in the extracellular space or bound to the outer leaflet of the plasma membrane [[10], [11], [12]]. Many galectins have been shown to be located outside of the cell, with galectins 1 and 3 perhaps being the best documented due to their well-established functions and their interactions with other extracellular proteins [13,14]. Interestingly, perturbations of the secreted levels of both annexins and galectins are associated with disease. For example, changes in the secreted levels of many annexin family members are associated with cancer [15]. Annexin A2 is a surface receptor for β2-glycoprotein I, a phospholipid-binding protein found in plasma known to be an autoantigen in the antiphospholipid antibody syndrome [16,17]. Galectins are broadly associated with infection and inflammation [18]. The level of galectin-3 in the serum is associated with cardiovascular disease, and is used as a biomarker to predict outcomes in heart failure [19,20]. These disease associations with altered secretion make it interesting to understand both the regulation of and the mechanism for secretion, as knowledge of this process this could ultimately have clinical applications.

## 2 Annexins

Annexins are phospholipid binding proteins forming a conserved family, with members expressed in animal and plant kingdoms. In most cases, their phospholipid binding activity is calcium (Ca^2+^) dependent [21]. Structurally, annexins are characterised by a conserved alpha-helical core domain, which functions as a calcium regulated membrane binding module, and a central hydrophilic pore, proposed to function as a calcium channel, as reviewed by Gerke and Moss[10]. Additionally, annexins have a unique N-terminal domain that confers individual annexin function [10,21]. A well established role of annexins is in membrane organisation, particularly in formation of membrane domains, via their binding activities to phospholipids [10,12,21].

Annexin’s extracellular localisation is evidenced by their function as receptors for extracellular proteins. In general, annexins outside the cell can act as receptors for serum proteases as well as regulators of cell migration and blood coagulation [[10], [11], [12]]. Annexin A2 has been described as a cell surface receptor for elements of the plasmin/plasminogen activator system present on the surface of endothelial cells, and for β2-glycoprotein I, as mentioned previously. Other ligands for annexin A2 on the cell surface include procathepsin B on cancer cells and 1α,25-Dihydroxyvitamin D_3_, a vitamin D analogue, on osteoblasts [22,23]. Extracellular annexins are also important in innate immunity, acting as neutralising agents by binding to pathogens [24].

The secretion of annexins must be regulated to ensure correct function. Annexin A4 and annexin A5 function outside the cell as anticoagulant factors via their Ca^2+^-regulated binding to phospholipids [10]. Annexin A5 plasma concentration is low and constant both during pregnancy and post-partem, whereas the level of annexin A4 increases shortly after delivery [25]. Moreover, antibodies directed against annexin A5, which inhibit its anticoagulant function, are found in patients with recurrent pregnancy losses with antiphospholipid syndrome [26]. This demonstrates that regulation of annexin secretion is essential in order to be expressed at the correct time in pregnancy.

### 2.1 Mechanisms of annexin secretion

Despite having described extracellular functions, the mechanisms of annexin secretion remain unclear. There are two major pathways currently proposed for the secretion of annexins: direct translocation across the plasma membrane and secretion via EVs. These pathways have been best studied using annexin A1 and A2, whereas little is known about the mechanisms for secretion of annexins A4 and A5. In most studies, only one annexin family member has been investigated, so it remains unclear whether the cellular mechanisms described apply to all family members.

#### 2.1.1 Direct translocation

Insertion of annexins into membranes has been demonstrated for several family members (annexin A1, A2, A4, A5 and A6), reviewed by Lizarbe et al. [21]. It has been further suggested that annexin oligomers can span the plasma membrane, as has been reported for annexin A12 [27]. Their proposed function as calcium channels also suggests that annexins are able to span membranes [28,29]. Additionally, several annexin family members are reported to induce leakage in liposomes, which is likely to be via insertion into the membrane [[29], [30], [31]]. Together, this suggests that it is possible that annexins do not require any other elements to cross the plasma membrane, pointing to an unfacilitated type I unconventional protein secretion pathway. However, many of these observations were made in the absence of calcium and at low pH; as such, it is unclear whether this would occur in vivo, and evidence for unfacilitated transport in cells and in vivo is limited.

We recently showed that annexin A2 and annexin A5 are able to translocate across membranes in liposomes and in cells. This translocation requires Ca^2+^-dependent binding to negatively charged phospholipids and lipid flipping activity (unpublished, preprint available [32]). Here there are similarities to FGF-2 translocation across membranes, requiring FGF-2 binding to lipids, with additional insight into the role of lipid remodelling in the translocation process [33]. Furthermore, we identified a phospholipid translocase called TMEM16F (anoctamin-6) which is required for lipid flipping activity in cells (unpublished, preprint available [32]). Our study provides the first evidence for a protein flipping mechanism dependent on lipid remodelling. However, this does not show the full mechanism: it remains unclear whether this translocation is facilitated or unfacilitated. It is also yet to be determined whether this is a general mechanism for unconventional secretion or if this is specific to annexin A2.

Alternatively, it has been suggested that annexin A1 is transported from the cytosol across the plasma membrane into the extracellular space by type II secretion by ABC-transporter proteins: ATP dependent membrane transporters responsible for transporting cargo such as ions, heavy metals, amino acids and sugars [34]. Certain bacterial ABC transporters are responsible for the secretion of larger molecules, including proteins, for example in the export of haemolysin A from *Escherichia coli* [34]. As such, it is possible that ABC transporters may also be responsible for the secretion of leaderless proteins in mammalian cells.

The first study to investigate a role for ABC transporters used glucocorticoid to stimulate annexin A1 externalisation in pituitary folliculo-stellate cells; ATP binding cassette A1 (ABCA1) transporter is readily detectable in these cells and co-localises with cell surface annexin A1 [35]. Additionally, annexin A1 externalisation was inhibited by glyburide, an ABC transporter inhibitor [35]. Based on this somewhat limited evidence, a role for ABCA1 in the secretion of annexin A1 was suggested [35]. A second group to report the involvement of ABC transporters for annexin secretion showed that annexin A1 secretion is inhibited by two ATPase inhibitors, vanadate and pervanadate [36]. This group used fresh mucosa from the rectum of rats experimentally inflamed by trinitrobenzenesulfonic acid, a tissue selected as it had been shown to actively secrete annexin 1 in concentrations easily detected [36,37]. Interestingly, in the second study glyburide did not inhibit annexin A1 secretion; the authors suggest that this discrepancy is due to differences in stimulation methods and the cell line used.

Although these studies give indirect evidence for the role of ABC transporters in annexin A1 secretion, there is no direct evidence to show that ABCA1 or any other ABC transporter is responsible for annexin A1 secretion. The above studies show that annexin A1 secretion is inhibited by ABC transporter inhibitors, but neither study conclusively shows that ABC transporters were involved, as it is unclear whether the inhibitors used are selective for ABC transporters exclusively. Therefore, it is possible that other transporters and/or processes are affected by the inhibitors used. Furthermore, contradictory data when using glyburide suggests that the mechanism for annexin A1 secretion differs depending on the stimulant and the cell line used.

#### 2.1.2 Extracellular vesicles

It is well known that annexins are commonly found in EVs [38]. In the cytoplasm, annexins are known to have important roles in membrane trafficking and endocytosis, therefore, it is likely that their association with EVs is due to their association with endosomes and MVBs, as reviewed by Futter and White [39]. Annexin A2 has been implicated in MVB formation and annexin A1 has been shown to be important for intraluminal vesicles budding within the MVB after epidermal growth factor stimulation [40,41]. These data support the idea that the annexins localise to EVs due to their membrane trafficking roles.

However, it has additionally been proposed that annexins are specifically packaged into exosomes for their secretion into the extracellular space. In this case, annexins would need to be released from secreted EVs to interact with extracellular ligands and bind to the cell surface. This makes the hypothesis controversial, as it remains to be shown whether EVs actually release their contents outside the cell. Adding evidence to the association between annexins and EVs, we have demonstrated that annexin A2 is detectable on the surface of cell line derived EVs [42]. Annexin A2 on the surface of exosomes is also thought to have functional consequences, particularly in the progression of breast cancer [43]. However, it is still unclear whether annexin A2 is specifically packaged onto the surface of EVs. It is possible that the annexin A2 detected on the extracellular face of EVs originates from the cell surface. In this case, annexin A2 would have to traverse at least one membrane: the plasma membrane, endosomal membrane or exosomal membrane. This is theoretically possible as our recent paper indicated that annexin A2 is able to cross membranes (unpublished, preprint available [32]). However, others have reported that EVs have inside-out protein localisation occurs [44], suggesting a specific mechanism for transporting proteins to the surface of EVs that remains to be identified.

Although there is no explanation as to how an EV pathway would mediate the trafficking of annexins to the cell surface, there is evidence to suggest that exosomes play a role in annexin secretion. As mentioned previously, interferon-γ stimulation increases cell surface translocation of annexin A2 in lung epithelial cells. Additionally, this increase can be inhibited by reagents that are thought to block exosome biogenesis [45]. These observations were reconciled into a pathway for trafficking annexin A2 into exosomes through the autophagosomal pathway, termed exophagy [46]. In this pathway, it is suggested that annexin A2 is trafficked into autophagosomes which then fuse with the MVB in a Rab11 dependent fashion [46]. The combined autophagosome and MVB, termed the amphisome, then fuses with the plasma membrane in a Rab8A and Rab27A dependent manner [46]. This process is also likely to be dependent on the initiation of autophagy, as annexin A2 secretion in exosomes decreased when autophagy protein 5 (ATG5) was silenced [46]. However, ATG5 has been shown to play a role in autophagy-independent processes, so it remains possible that this effect does not involve autophagy pathways [47].

While it is clear that interferon-γ stimulation increases both the amount of annexin A2 on the cell surface and the amount associated with EVs, it remains unclear if these two processes are directly linked. It has not convincingly been shown that the inhibition of exophagy results in a decrease in annexin A2 at the cell surface. Furthermore, whether this process occurs under unstimulated conditions remains unknown; it may be specific to interferon-γ stimulation.

### 2.2 Regulation of annexin secretion

Various compounds have been shown to alter annexin secretion, and some mechanisms for this regulation have been documented. Many of these mechanisms involve Ca^2+^. The corticosteroid dexamethasone induces release of annexin A1 from the human immature lymphoblastic CCRF-CEM cell line [48]. This release is Ca^2+^-dependent; Ca^2+^ influx triggers annexin A1 cleavage, which is required but not sufficient for secretion [48]. Glutamate has also been associated with annexin A2 secretion in a process dependent on N-methyl-D-aspartate (NMDA) receptor activity, a Ca^2+^ channel facilitating Ca^2+^ influx, in the transformed mouse photoreceptor cell line 661W [49].

The Ca^2+^-binding protein p11 is implicated in regulation of surface translocation of annexin A2 by both interferon-γ and thrombin [45,50]. Annexin A2 binds directly to p11 in response to Ca^2+^ influx [51]. Interferon-γ enhances surface translocation of annexin A2 via activation of the JAK2/STAT1 signal pathway, dependent on the interaction between annexin A2 and p11, in lung epithelial cells [45]. Thrombin also increases annexin A2 secretion in a p11-dependent manner in umbilical vein endothelial cells (HUVECs) [50]. Together, this suggests that trafficking of annexin A2 across the plasma membrane is controlled by p11.

A common trigger of unconventional secretion is cellular stress, including stress from infection and inflammation [[52], [53], [54]]. Inflammation is characterised by a localised emigration of neutrophils and other leukocytes from the blood to inflamed tissue, which can be delayed by glucocorticoids. Annexin A1 has been shown to regulate this glucocorticoid effect [55]. Neutrophils adhering to activated endothelium are able to externalise annexin A1 via an unknown mechanism, resulting in a decrease of neutrophil extravasation caused by annexin A1 binding to integrins and G protein-coupled receptors (GPCRs) [[56], [57], [58]]. Because the mechanism of annexin secretion is unknown, it remains possible that the extracellular annexin A1 originates from lysed cells during inflammation. However, temperature stress can also lead to annexin A2 secretion in endothelial cells; this does not cause cell death or cell lysis and is dependent on both p11 and phosphorylation of annexin A2 [59]. A recent report showed the role of macrophage activation and polarisation in annexin secretion [60]. P2X7R stimulation of macrophages releases annexins A1, A2 and A4 independently of the macrophage polarisation state, suggesting a potential role for P2X7R during resolution of the inflammation [60]. Finally, stress-activated caspase-1 has been implicated in annexin A2 secretion, verified by siRNA knockdown [61]. Caspase-1 has been proposed to be a more general regulator of unconventional protein secretion, although evidence for this is limited.

## 3 Galectins

Galectins are galactose-specific lectins: proteins with conserved carbohydrate recognition domains which bind β-galactosides via their carbohydrate recognition domain (CRD) [62]. They can be categorised into three groups: prototypical galectins, which have one CRD; tandem repeat galectins, with two CRDs with different specificities to each other; and the chimeric group consisting only of galectin-3, which has one CRD and a large, flexible N-terminal domain (NTD) [13]. Most of the prototypical galectins have been shown to exist as homodimers [63], and tandem repeat galectins are bivalent by definition, with each galectin in this group having two CRDs with different specificities to each other [64,65]. Galectin-3′s NTD confers multivalent properties via self association [66], both via NTD-NTD interactions and NTD-CRD interactions [67]. This multivalent property of galectins allows the formation of galectin-glycan lattices, as reviewed by Rabinovich et al. [63].

Many studies have found that galectins have different and sometimes opposing functions inside and outside of the cell, particularly for galectin-3, one of the most well-studied galectins [13,[68], [69], [70], [71]]. For example, galectins such as galectin-1 and galectin-3 are able to induce apoptosis outside the cell, thought to be mediated by their interactions with glycans [72,73]. In contrast, galectin-3 inhibits apoptosis inside the cell, likely via protein-protein interactions [68,71]. Outside the cell, galectins are involved in cell-cell and cell-matrix interactions via interactions with proteins including laminin and fibronectin [71]. They have highly diverse functions, including in cell adhesion, epithelial homeostasis and as chemoattractants in the immune system [18,70,[74], [75], [76]]. As the specific functions of galectins are dependent on both the cell type they are expressed in and on their cellular or extracellular localisation, their secretion must by tightly regulated in order for them to function correctly.

### 3.1 Mechanism of galectin secretion

Mechanisms for the export of galectins are not well understood. Most of the work elucidating a mechanism for secretion has been performed using galectin-1 and galectin-3, so this section will primarily focus on these proteins. Work on the mechanism has pointed to different routes for secretion, but it is possible that galectins are able to make use of more than one pathway [77].

#### 3.1.1 Oligomerisation of galectins may be required for secretion

It has been reported that deletion of the first twelve residues of galectin-3′s N-terminal domain (NTD) blocks the export of galectin-3 into the cell supernatant [78]. That the NTD is required for secretion is a surprising finding as all members of the galectin family that are secreted use an unconventional pathway, yet the extended NTD is unique to galectin-3. Delacour et al. propose that it is not the NTD itself but rather oligomerisation that is necessary for galectin-3 secretion, as this is the domain that is responsible for oligomerisation of galectin-3 [79]. It is a possibility that oligomerisation is universally required for galectin secretion, although there are no studies that have investigated this specifically. Although there are galectins which predominately exist as monomers, many of these have more recently been shown to also exist as dimers, such as galectin-7 and galectin-8 [14,80,81]. Alternatively, as this study only looked at galectin-3 in the supernatant [78], it is possible that the galectin-3 NTD mutant is in fact secreted but remains tightly bound to the cell surface. This is unlikely, as the NTD acts to increase binding affinity to galactomannans, and would be expected to increase avidity to galectin-3 ligands [82]. However, whether the mutant is present on the cell surface needs to be determined given that the mechanism by which the NTD alters galectin-3′s binding functions is unknown [82]. It could also be that different galectins are secreted by different pathways in response to different signals, as suggested by Barondes et al. [76].

#### 3.1.2 Direct translocation

Previous work has suggested that galectin-1 uses the same route as FGF-2 for its secretion, which is the most-studied unconventionally secreted protein that uses the direct translocation pathway. in vitro studies have shown that both proteins are imported into inside-out vesicles derived from the plasma membrane, implying that they are both directly translocated across the plasma membrane [83]. Recent work has highlighted the importance of the phospholipid PI(4,5,)P_2_ in the unconventional secretion of FGF-2. PI(4,5)P_2_ is required for recruitment of FGF-2 to the plasma membrane and FGF-2 oligomerisation to form a pore for transport across the membrane [33,84,85]. It is possible that the FGF-2 mechanism for secretion represents a more widely applicable unconventional secretion mechanism, as IL-1β also uses PI(4,5)P_2_dependent pore formation for secretion, but the role of PI(4,5)P_2_ has not been studied for the galectins [86]. It has also been found that heparin sulfates on the cell surface act as counter receptors to ensure FGF-2 is translocated in one direction [85,87]. If FGF-2 and galectins are exported from cells in the same way, it could be that glycans on the cell surface act in a similar way, as previous work with galectin-1 suggested that glycans are required for secretion [88]. In direct contrast to this, CHO cell with glycosylation defects are still able to secrete galectin-1 [89]. Furthermore, we recently showed that galectin-3 can be exported from cells when glycosylated proteins are not available; galectin-3 was detected only in the cell supernatant as it has no binding partners on the cell surface [90]. If there is a common pathway for all galectins’ secretion, a requirement for glycans seems unlikely, as it has been demonstrated that both galectin-1 and galectin-3 can be secreted in their absence.

Interestingly, galectin-3 has been shown both to interact with lipids found in the plasma membrane and to spontaneously move across the lipid bilayer [91]. This was shown in both live cells and membrane models; as the membrane models did not contain membrane proteins, it is possible that no additional machinery would be required for the export of galectin-3, pointing to type I unfacilitated direct translocation as a pathway. Furthermore, it is thought that both galectin-1 and galectin-3 accumulate on the cytoplasmic side of the plasma membrane before being secreted; this has been clearly demonstrated for galectin-1 but has been assumed for galectin-3 [92,93]. Although this accumulation would be required for type I direct translocation, in itself this does not exclusively implicate direct translocation as a mechanism, as release in microvesicles budding from the plasma membrane would also involve this step.

#### 3.1.3 Release in extracellular vesicles

There is evidence for the release of galectins from cells in EVs, either being released in microvesicles formed from membrane blebbing, or from exosomes that originate from MVBs. Overall, these routes are not well defined, with the question of how galectins are released from these vesicles into the extracellular space remaining unanswered.

Most of the work regarding galectin release in EVs implicates microvesicles. In cultured mouse muscle cells, galectin-1 has been shown to accumulate in concentrated patches underneath the plasma membrane, where microvesicles loaded with galectin-1 then form and are released into the medium [93]. Secretion in microvesicles has also been demonstrated for a galectin-3 chimera protein: a fusion of galectin-3 and a Lck segment [92]. The Lck segment directs Lck, or in this case the chimera, to the cytoplasmic side of the plasma membrane. Alone, the Lck segment increases traffic to the membrane but does not affect secretion, demonstrated by the fact that the segment fused to the CRD of galectin-3 was not secreted [92]. This is also in accordance with the finding that deletion of the first twelve residues of galectin-3′s NTD blocks galectin-3 export into the cell supernatant [78]; the galectin-3 chimera protein was also not secreted without galectin-3′s NTD [92]. Although this is good evidence for release of galectin3 in microvesicles, this model relies on the assumption that endogenous galectin-3 is able to accumulate under the plasma membrane.

On the cell surface, galectin-3 forms clusters with glycosphingolipids and glycosylated proteins, which causes mechanical strain that leads to formation of clathrin-independent carriers inside cells [94]. As these vesicles form in the opposite direction to microvesicles, this is not directly relevant to secretion, but does demonstrate that galectin-3-glycan clusters are able to induce membrane bending. Adding to this, other work has shown that a mechanical stimulus, of either scraping or trypsinisation to suspend cells, induces galectin-3 secretion; here it was suggested, although not demonstrated, that a mechanical stimulus might induce secretion in microvesicles [95]. It is theoretically possible that this mechanism invoked by mechanical strain to form internal vesicles could also work in the opposite direction and lead to microvesicle formation. Again, galectin-3 would need to accumulate underneath the plasma membrane for this to take place. While it has been shown that galectin-1 accumulates underneath the plasma membrane [93], this remains to be demonstrated for endogenous galectin-3.

Additional work supports a model of release either via exosomes or via lysosomes, although this may exclusively be as part of a stress response. The induction of autophagy by CPP recruits galectin-3 to the late endosome and/or lysosomes in HEK293 cells, although it has not been determined whether galectin-3 is inside or on the surface of these vesicles [96]. MVBs are a type of late endosome, so this may also implicate the exosomal pathway. Furthermore, galectin-3 and galectin-4 have been shown to accumulate in endosomes [97,98]. How galectins get into these vesicles remains an unanswered question. One possible route is via damaged vesicles; galectins 1, 3, 8 and 9 all localise to damaged lysosomes and remain there for several hours, regardless of whether the cell dies or recovers [99]. Here galectins function as sensors of lysosomal damage [100]. If these damaged lysosomes do represent part of a secretion pathway, only lysosomes that recover from damage could be involved in secretion. Regardless of how galectins enter lysosomes or endosomes, this pathway cannot represent a universal route for secretion as galectins are secreted from cells at a basal level; however, it may be important for secretion induced by cell stress.

### 3.2 Regulation of galectin secretion

Little is known about the regulation of galectin secretion, and it is unclear whether regulation is universal as only galectin-3 and galectin-9 have been well-studied. Two key regulators of galectin3 secretion identified are calcium and serum. The calcium ionophore A23187, which induces a Ca^2+^ influx to the cytosol, increases transport of galectin-3 into the extracellular space from Madin-Darby Canine Kidney cells and from Baby Hamster Kidney cells [101,102]. Serum free media decreases galectin-3 secretion [102], while the serum protein fetuin can stimulate rapid export of galectin-3 from breast cancer cells [103]. Work in the Jurkat T cell line has shown that phorbol 12-myristate 13-acetate (PMA), an activator of protein kinase C (PKC), induces both an overall upregulation of galectin-9 mRNA and an increase in galectin-9 on the cell surface [104]. However, further work is needed to clarify the role of PKC here, as a PKC inhibitor only makes a slight difference to galectin-9 levels [104]. Additionally, PKC is involved in many signalling pathways in the cell and it is unknown which of these pathways would be involved in galectin-9 secretion. Furthermore, another study found that PMA treatment of human THP1 cells induces an increase in galectin-3 mRNA and protein levels of galectin-3 both in the medium and in exosomes, but this study attributes the change to the role of PMA as an inducer of NADPH activity [105]. While these studies are in accordance with each other in that they show PMA upregulates galectin, they both lack any direct evidence for the mechanism by which this drug effects the change.

Another potential regulator of galectin secretion is caspase-1. Both galectin-3 and galectin-1 were identified as possibly being regulated by stress-activated caspase-1, alongside annexin A2 and other unconventionally secreted proteins. However, regulation of galectin secretion by caspase-1 has not been specifically verified [61]. Stress in general, particularly as a result of infection, is implicated in galectin secretion. A number of viral infections are associated with an increase in galectin levels in serum and plasma, reviewed by Merani et al. [106]. A recent study showed that secretion of galectin-9 increases upon dengue viral infection in human THP-1 cells, suggested to function as a protective mechanism for cells by limiting viral attachment to surface glycans [107].

Other regulators of extracellular galectin levels have been identified, but these do not directly affect galectin secretion. For example, galectin-3 expression can be modulated by the hormones 17β-oestradiol, progesterone and human chorionic gonadotropin, which all stimulate translation of galectin-3 and lead to increased secretion of galectin-3 from the embryonic cell line BeWo [108]. Outside the cell, matrix metalloproteinases (MMPs) cleave galectins, so extracellular levels of galectin-3 decrease when MMP2 and MMP9 increase [109,110]. Conversely, broad-range MMP inhibitors suppress the release of galectin-9 from Jurkat cells, possibly suggesting MMPs are needed to release galectin-9 from the cell surface [104].

## 4 Conclusions

Galectins and annexins are functionally unrelated, but both families have extracellular functions and lack an N-terminal signal sequence to direct them through to the ER for secretion. Although in both cases much is left to be determined about how they are unconventionally secreted, there are some common themes in what little is known.

In terms of the mechanism for secretion, there is some evidence to suggest that oligomerisation is important for both of these protein families to be secreted. For galectin, this is mostly an implicit suggestion, based on the evidence that the N-terminal domain of galectin-3 is required both for its oligomerisation and secretion, yet is unique to the only chimeric galectin. This is very limited evidence if it is used to generalise to all galectins, and is primarily based on the assumption that all of the galectins would follow the same pathways for secretion. Although this may well be a reasonable assumption, there is no direct evidence to support it. For the annexins, it has been shown that annexin A12 oligomers span membranes, but again there is no direct evidence to show that other annexins do this. It is likely that in both cases oligomerisation would be part of a direct translocation mechanism. If oligomerisation is indeed involved for both annexin and galectin secretion, this may suggest that it is a common feature to unconventional secretion more generally, as FGF-2 also needs to oligomerise as part of its secretion [33].

There is also evidence for both annexins and galectins being associated with EVs. Again, the story is not complete here. Often associations have been shown, but details remain undiscovered. Both galectins and annexins localise to vesicles, but there are key questions remaining: whether these proteins are specifically packaged into vesicles, and how they are released from EVs. These questions are relevant to any unconventionally secreted protein proposed to use EVs for secretion. The question of release remains contentious as it is unknown whether EVs release their contents.

The regulation of galectin and annexin secretion seems to have few shared aspects, although many studies have used drugs to investigate regulation, so it is hard to know what comes from specific action of the protein intended to be targeted and what comes from off-target drug effects. A comparison of the known regulators is shown in Table 1. One common theme in regulation is calcium ions. For both proteins, an influx of Ca^2+^ is associated with an increase in secretion. This may implicate an EV pathway, as both microvesicles and exosomes are released in response to an increase in intracellular calcium ions [111,112]. Additionally, both galectin and annexin secretion is upregulated by cell stress, and stress-induced caspase-1 is proposed to regulate both proteins’ secretion. However, stress cannot be the only regulator of secretion as both proteins are secreted at a basal level.

**Table 1:** Evidence for regulation of galectin and annexin secretion.

Regulator	Effect	Evidence	Result	Refs.
Calcium ion influx	Increases galectin and annexin secretion	Calcium ionophore A23187 treatment of kidney cells	↑ galectin-3 secretion	[101,102]
		Corticosteroid-induced calcium ion influx triggers annexin A1 cleavage	↑ annexin A1 secretion	[48]
		Glutamate stimulation of NMDA receptor, inducing calcium ion influx	↑ annexin A2 secretion	[49]

p11	Increases annexin A2 secretion	Interferon-γ treatment of lung epithelial cells	↑ annexin A2 secretion	[45]
		Thrombin treatment of umbilical vein endothelial cells	↑ annexin A2 secretion	[50]
		p11 knockdown in mouse endothelial cells	↓ annexin A2 secretion	[59]

Stress	Generally increases unconventional secretion	Reviewed by Merani et al.	↑ galectin secretion	[106]
		Dengue viral infection of THP-1 cells	↑ galectin-9 secretion	[107]
		Neutrophils externalise annexin A1 during extravasation	↑ annexin A1 secretion	[[56], [57], [58]]
		Temperature stress of endothelial cells	↑ annexin A2 secretion	[59]

Caspase-1	Potential stress-activated regulator of some unconventionally secreted proteins	Galectin-1, galectin-3 and annexin A2 identified in a mass-spectrometry-based screen to identify secreted proteins regulated by caspase-1		[61]
		Knockdown of caspase-1 in Human primary fibroblast	↓ annexin A2 in supernatant	[61]

P2X7R	Increases annexin secretion	P2X7R stimulation of macrophages	↑ annexin A1, A2 and A4 secretion	[60]

Serum	Increases galectin-3 secretion	Cells cultured in serum free medium	↓ galectin-3 secretion	[102]
		Fetuin treatment of breast cancer cells	↑ galectin-3 secretion	[103]

PMA	Increases galectin-9 secretion	PMA treatment of Jurkat T cell line	↑ galectin-9 on cell surface	[104]
		PMA treatment of THP-1 cells	↑ galectin-9 in medium, exosomes	[105]

For both proteins, there is evidence implicating more than one pathway. A possible explanation for this is that different pathways could either be utilised by different members of each family, or used in different situations by the same protein. There is a precedent for the latter in the case of IL-1β. Upon inflammation, IL-1β is secreted by a type I pathway through plasma membrane pores, whereas under starvation it uses secretory lysosomes in a type III pathway [[113], [114], [115]]. It is therefore possible, for example, that annexins are transported across the plasma membrane at steady state and use EVs in stress conditions such as stimulation by interferon-γ.

Overall, although the extracellular localisation of annexins and galectins has been well-established, many questions surrounding the unconventional secretion of galectins and annexins still need to be resolved. There is evidence for both galectins and annexins being secreted via direct translocation and using a vesicle-based pathway, but it is unclear how these separate pathways relate to each other. Studies into the regulation of secretion often rely on drugs which may have multiple effects, so few conclusions on the mechanism for regulation can be confidently drawn. This review highlights that there is a clear need for more work to investigate the mechanism and regulation of galectin and annexin secretion.

## Funding Statement

This work was supported by Wellcome Trust Strategic Award [100574/Z/12/Z], MRC Metabolic Diseases Unit [MRC_MC_UU_12012/5], BBSRC Future Leader Fellowship and the Isaac Newton Trust/Wellcome Trust ISSF/University of Cambridge joint research grant for SES and KM, Wellcome Trust PhD Studentship to SJP.
